# Determination of ROS Scavenging, Antibacterial and Antifungal Potential of Methanolic Extract of *Otostegia limbata* (Benth.) Boiss.

**DOI:** 10.3390/plants10112360

**Published:** 2021-11-02

**Authors:** Huma Mehreen Sadaf, Yamin Bibi, Muhammad Ishaque, Sobia Nisa, Abdul Qayyum, Naila Safdar, Zahid Hussain Shah, Hameed Alsamadany, Gyuhwa Chung

**Affiliations:** 1 Department of Botany, PMAS-Arid Agriculture University Rawalpindi, Rawalpindi 46000, Pakistan; humasadaf00@yahoo.com (H.M.S.); dryaminbibi@uaar.edu.pk (Y.B.); mishaque270@gmail.com (M.I.); 2 Department of Microbiology, The University of Haripur, Haripur 22620, Pakistan; sobia@uoh.edu.pk; 3 Department of Agronomy, The University of Haripur, Haripur 22620, Pakistan; 4 Department of Biotechnology, Fatima Jinnah Women University, Rawalpindi 46000, Pakistan; nailahussain@fjwu.edu.pk; 5 Department of Plant Breeding and Genetics, PMAS-Arid Agriculture University Rawalpindi, Rawalpindi 46300, Pakistan; shahzahid578@hotmail.com; 6 Department of Biological Sciences, King Abdulaziz University, Jeddah 21589, Saudi Arabia; halsamadani@kau.edu.sa; 7 Department of Biotechnology, Chonnam National University, Yeosu 59626, Chonnam, Korea

**Keywords:** antimicrobial, DPPH, crude extract, medicinal plants

## Abstract

Wide spectrum medicinal significance augments plant utilization as the primary source of significant pharmaceutical agents. In vitro investigation of antioxidant and antimicrobial activity highlights the therapeutic potential of *Otostegia limbata*. Methanol extract of the plant (MEP) shows considerable dose dependent antioxidant ability at six concentrations (7.81 µg/mL to 250 µg/mL) in 2.2-diphenyl-1-picrylhydrazyl (DPPH) assay, phosphomolybdate assay (PMA) and reducing power assay (RPA). The plant capability to scavenge free radicals in the mixture ranged from 37.89% to 63.50% in a concentration-dependent manner. MEP was active against five tested bacterial strains in the agar-well diffusion method. *Staphylococcus aureus*, gram-positive bacteria was found to be most susceptible followed by *S. epidermidis* with 18.80 mm and 17.47 mm mean zone of inhibition. The mean inhibition zone against gram-negative strains *Klebsiella pneumonia*, *Pseudomonas* spp. and *Escherichia coli* were 15.07 mm, 14.73 mm, and 12.17 mm. MEP revealed potential against *Alternaria* spp. and *Aspergillus terreus* fungal strains evaluated through agar-tube dilution assay. *Aspergillus terreus* was more sensitive than *Alternaria* spp. with an average 78.45% and 68.0% inhibition. These findings can serve as a benchmark for forthcoming scrutiny such as bioactive components discovery and drug development.

## 1. Introduction

Exploration of the therapeutic potential of plants indicates the presence of antimicrobial principles. Renewed interest in plant antimicrobials has emerged in the last 20 years, possibly due to growing drug resistance of human pathogens, over and above the undesirable side effects of synthetic antibiotics [1]. Many bacteria, such as *Staphylococcus aureus* or *enterococci* are resistant to antibiotics such as methicillin or vancomycin. The development of potential multidrug resistance in pathogens is a key motivator for finding novel molecules or groups of compounds that can be used in pharmaceuticals without the toxic effects of synthetic chemical compounds [2]. Ultimately, research should be focused in order to discover as much potentially appealing data as possible, together with negative and positive interactions with general antibiotics and so forth. Such findings could further improve the use of medicinally important plants, their extracts, or other natural products, in any form: alone or in combination with antibiotics. The researchers developed their interest in biologically potent compounds which are isolated from different plant species meant for the removal of pathogenic microorganisms due to resistance built into microorganisms against antibiotics [3].

In plants, harsh environmental conditions, for example salt constraint, cause improved production and accumulation of the reactive oxygen species (ROS), initiating cellular damage, severe metabolic disorders, and senescence pathways [4]. In living beings, different ROS are able to form in diverse methods. ROS have been involved in over 100 ailments, counting heart disease, malaria, stroke, acquired immunodeficiency syndrome, arteriosclerosis, cancer, and diabetes [5]. Plants are well-known for their capability to withstand unfavorable environments and quench toxic ROS, as they are equipped by means of powerful antioxidant systems that involve both non enzymatic and enzymatic components [6]. Antioxidant compounds are able to delay oxidation of lipids and prevent oxidation of other molecules through inhibition of initiation and proliferation of oxidative chain reaction. Therefore, they can prevent and repair cellular damage triggered by oxygen species [7]. All these compounds are considered as hydrogen donors, chelating agents of prooxidants, reducing agents, free radical scavengers, and singlet oxygen quenchers [8]. In recent times, there is a huge interest in measurement as well as exploitation of plant antioxidants for scientific research and industrial uses (dietary, cosmetic, and pharmaceutical). This is primarily as a result of strong biological activity, surpassing those of synthetic antioxidant components which are active as carcinogens and considered to be its promoters [9]. Thus, a need exists for powerful, economic, natural, and safe antioxidants in the replacement of synthetic antioxidants [10].

The plant kingdom is considered to be the best source of medication for a variety of pains and ailments. For this reason, medicinally important plants have played key roles in maintenance of health worldwide. Higher plants and their natural products are a vital source of effective therapeutic agents. Thus, countless research groups are presently involved in the screening of plants for their diverse biological activities. *Otostegia limbata* (syn. *Ballota limbata*) belongs to Lamiaceae (Labiatae) family and genus Otostegia possesses twenty species distributed in the Mediterranean region. Only three species of genus Otostegia, i.e., *O. persica*, *O. aucheri*, and *O. limbata* are reported from Pakistan. *Otostegia limbata* (Benth) Boiss is a valuable bioactive plant extensively distributed in hilly regions of Khyber Pakhtoonkhawa (KPK) and Punjab provinces of Pakistan. Locally it is known as Spina ghazai, Koi booi, Chota kanda, Bui, Chittie bootie or Chitta jand. It is a bushy, slender, branched, pubescent, spiny, and small shrub up to almost 2 ft tall while its flowering period is April–June. Its stem is woody, branched, erect, spiny, with gray and whitish bark. Small, dentate, oblanceolate in shape, not entirely divided leaves with short petiole and spiny bracts. Flowers of the plant are long, pale yellow to orange throated, prominently bilabiate with straight upper-lip and spread lower-lip and present in axillary clusters [11]. 

*O. limbata* is well known for many traditional medicines intended for several purposes. Juice of the plant is effective for the treatment of wounds, as an ophthalmic medication, and a valuable product to treat bleeding gum problems in children. Crushed fresh leaves with low amounts of water in the form of an extract are used locally to treat different types of eye infections [12]. Antimicrobial agents are extremely vital in reducing the large-scale burden of infectious maladies. People indigenous to the area in which it grows have been consuming numerous plant species as conventional remedies for many years, however there has been a paucity of information regarding in vivo and in vitro efficacy. Yet, there are inadequately detailed or thorough investigations into the potential role of the plant as an antimicrobial and therapeutic entity for MDR bacteria and pathogenic fungi [13]. Considering extensive potentiality of the plant as an antimicrobial drug source, this analysis was aimed to examine its in vitro antioxidant, antibacterial, and antifungal activity against the most common contagious pathogens. To this end, the main objectives of this study were (i) to estimate antioxidant activity using various tests, (ii) and to assess antimicrobial capacity against different human pathogenic microbial (bacterial and fungal) strains. 

## 2. Materials and Methods

### 2.1. Preparation of Extract

*Otostegia limbata* aerial parts (leaves branches and flowers) were harvested in May 2013 from Margalla hills, Islamabad. Plant specimen was submitted to the Herbarium of PMAS Arid Agriculture University Rawalpindi for future reference. Cold maceration technique was used to prepare the plant extract. Five grams of dried powdered plant material were soaked in 50 mL of methanol in an Erlenmeyer flask for almost 2–4 weeks and the mixture filtered by using Whatmann filter paper No. 1. Filtrate was concentrated by evaporation of the solvent through a rotary evaporator. The same process was repeated many times to obtain the maximum amount of extract. The obtained extract (2.74 g) was stored for different assays at 2 to 6 °C in the phytochemistry lab in the PMAS Arid Agriculture University Rawalpindi.

### 2.2. Antioxidant Assays

#### 2.2.1. DPPH Radical Scavenging Activity Assay

The DPPH assay according to the method [12] was carried out with some alterations. DPPH in methanol was used as stock and diluted to obtain 0.980 ± 0.02 absorbance noted at 517 nm. Stock solution (3 mL) and samples (7.81 µg/mL–250 µg/mL) were mixed and shaken vigorously followed by 15 min incubation time period. Positive reference in assay was ascorbic acid and percentage scavenging effect calculated through following equation:

Scavenging effect:

% = [(*A*_0_ − *A*_1_)/*A*_0_] × 100 (1)

where *A*_0_ was control absorbance and *A*_1_ was sample absorbance recorded in nm by spectrophotometer.

#### 2.2.2. Phosphomolybdate Assay

The antioxidant potential was assessed through procedure [14] with minor changes. Sample and reagent solution (28 mM sodium phosphate, 0.6 M sulphuric acid and 4 mM ammonium molybdate) was mixed covered properly with aluminum foil, incubated at 95 °C in water bath and at room temperature mixture was allowed to cool. Measurement of absorbance at 765 nm against blank and ascorbic acid was utilized for comparative analysis as standard.

Total antioxidant capacity (%) = [(Abs. of control − Abs. of sample)/(Abs. of control] × 100(2)

#### 2.2.3. Reducing Power Assay

The reducing potential of *O. limbata* was revealed by the process of Jindal and Mohamad [15]. Potassium ferricyanide, phosphate buffer (0.2 M) and extract solution were mixed in equal volumes (2 mL). The mixture was incubated at 27 °C followed by addition of trichloroacetic acid (2 mL), ferric chloride (0.4 mL) and distilled water. Absorbance was recorded at 700 nm and raise in the value of absorbance is indication of more reducing potential.

### 2.3. Antibacterial Assays

Agar well diffusion method [16] with some minute alterations was used to investigate antibacterial potential. Autoclaved nutrient agar medium (20 g/L; pH: 7) was poured into Petri plates and overnight refreshed bacterial culture (10 mL) was inoculated. Wells (8 mm) were made and labeled and plant extract (20 mg/mL in DMSO), Cefotaxime (positive control), and DMSO (negative control) were poured into their respective wells. Inhibition zone was measured after 24 h incubation period at 37 °C and percentage inhibition was determined by using following formula:

Inhibition

% = [(TS – SC) / PC] × 100(3)

where TS–Test sample; SC–Solvent control; PC–Positive control

### 2.4. Antifungal Assay

Antifungal activity was estimated using agar tube dilution assay [17] with some modifications. Sabouraud dextrose agar (32.5 g/500 mL) was autoclaved and methanolic extract of plant (20 mg/mL in DMSO), Fluconazole and DMSO (positive and negative control) was poured into the media. Vigorously shaken test tubes were placed in oblique positions to create slant and allowed to solidify at room temperature. The tubes were inoculated with inoculums taken from 5 to 7 days old fungal culture and incubated for a week at 28 °C. Linear growth (mm) was quantified and growth inhibition (%) calculated by the following formula:

Inhibition

% = [100 − Linear growth in sample/Linear growth in control] × 100(4)

## 3. Results

### 3.1. Evaluation of Antioxidant Potential

**Figure 1:**
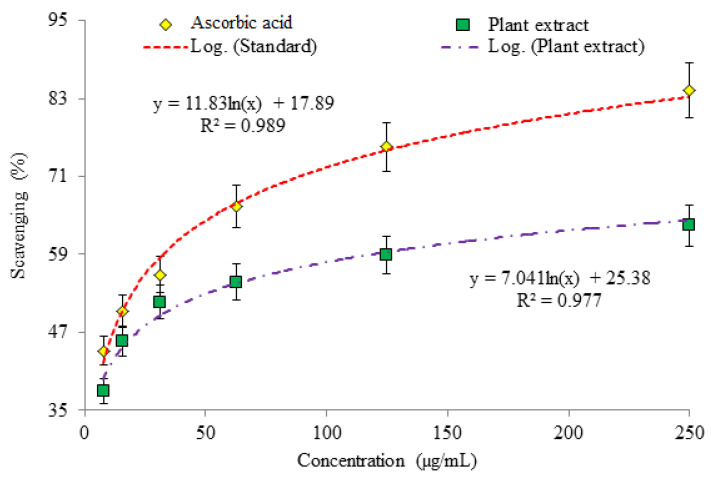
Percentage inhibition of ascorbic acid and plant extract in DPPH assay.

**Figure 2:**
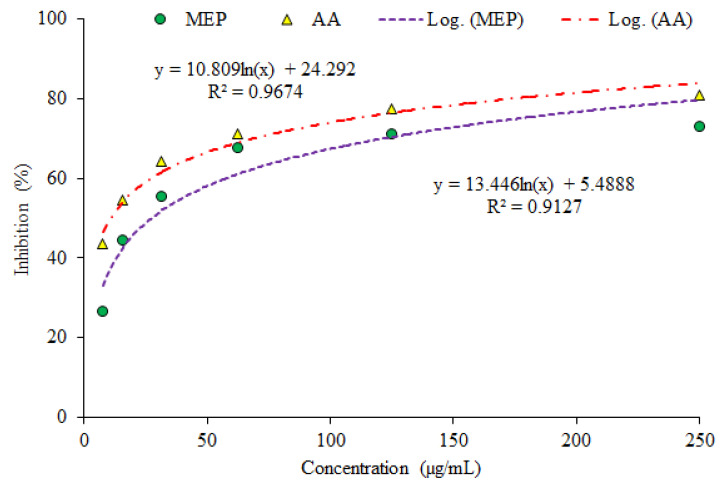
Percentage scavenging at various concentrations in phosphomolybdate assays (PMA) (MEP–Methanol extract of plants; AA–Ascorbic acid).

**Figure 3:**
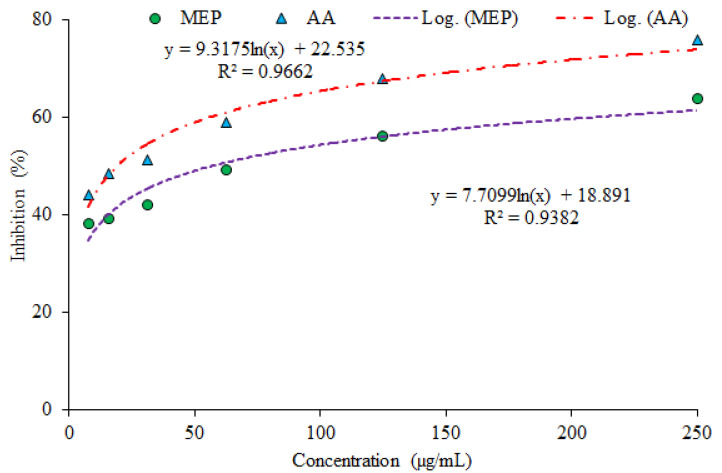
Percentage scavenging at various concentrations through reducing power assay (RPA) (MEP–Methanol extract of plants; AA–Ascorbic acid).

The outcomes of the investigation showed the considerable antioxidant potential as evaluated by three assays (DPPH assay (Figure 1), phosphomolybdate assay (PMA; Figure 2) and Reducing power assay (RPA; Figure 3). 

**DPPH assay:** Absorbance of *O. limbata* extract observed by DPPH (2, 2-diphenyl-1-picrylhydrazyl) assay on a range of concentrations to determine scavenging potential. The lowest absorbance of methanol extract of plant (MEP) was observed on 0.357 nm and for ascorbic acid (AA) 0.153 nm was recorded at the highest (250 µg/mL) concentration. The absorbance of MEP increased in the range of 0.402 nm–0.608 nm by diluting the sample in five different concentrations (125 µg/Ml–7.81 µg/mL) while observed absorbance of AA ranged from 0.239 nm–0.547 nm (Table 1).

**Phosphomolybdate assay:** The percentage inhibition value directly correlates with concentration of sample in phosphomolybdate assay (PMA). The highest percentage inhibition of MEP and AA was 73.22% and 80.77% at 250 µg/mL concentration. The percentage inhibition of MEP was in the range of 71.34% to 26.59% while percentage inhibition of AA was observed at 77.34–43.44% at concentrations ranging from 125 µg/mL to 7.81 µg/mL. Variations in percentage inhibition are highly dependent over a wide concentration range with antioxidant samples and standard (Figure 2). 

**Reducing power assay:** The reductive capability of plant extract assessed by reducing power assay (RPA) was also significant, with upper limit of scavenging at 63.94% for MEP and 76.02% for AA. Radical scavenging potential of MEP and AA was greatly influenced by plant as well as ascorbic acid concentrations. Figure 3 illustrates the reductive capabilities of MEP compared with AA. All the MEP concentrations tested were found to be active and statistically significant (*p* < 0.05) outputs were observed. The current findings authenticate the very strong aptitude of the plant for antioxidant activity.

IC_50_ values of the methanol extract of the plants and ascorbic acid used as standard were calculated through the regression line equation against DPPH antioxidant assay, phosphomolybdate assay and reducing power assay. It was found to be considerably low in AA, indicating its great antioxidant potential. The IC_50_ value of MEP also indicates antioxidant ability of plants in the three assays (Table 2)

### 3.2. Antibacterial Potential

The considerable antibacterial potential of MEP was observed against various bacterial strains. *Staphylococcus aureus* was the most susceptible bacterial strain to MEP followed by *S. epidermidis.* The highest inhibition zone of 18.80 mm was detected against *S. aureus,* followed by 17.47 mm zone against *S. epidermidis* (Table 3). Besides these, two bacterial strains were also receptive to standard commercial Cefotaxime (antibiotic drug) and mean growth inhibition zones observed were 19.07 mm and 17.83 mm, respectively. Selected *Staphylococcus* bacterial strains were the most susceptible strains among the other five chosen strains by their observed percentage inhibition of 98.60% and 97.94%, respectively. The least inhibition among the five chosen bacterial strains was against *Escherichia coli,* with the lowest mean inhibition zone value of 12.17 mm in diameter. *E. coli* also reveals susceptibility to commercial Cefotaxime with a recorded mean inhibition zone of 15.10 mm (Table 3). The inhibition percentage shown by the MEP against *E. coli* was found to be 80.57%. 

One-way analysis of variance showed that MEP had significant variances as antibacterial agent against the five different tested strains (Table 4).

**Figure 4:**
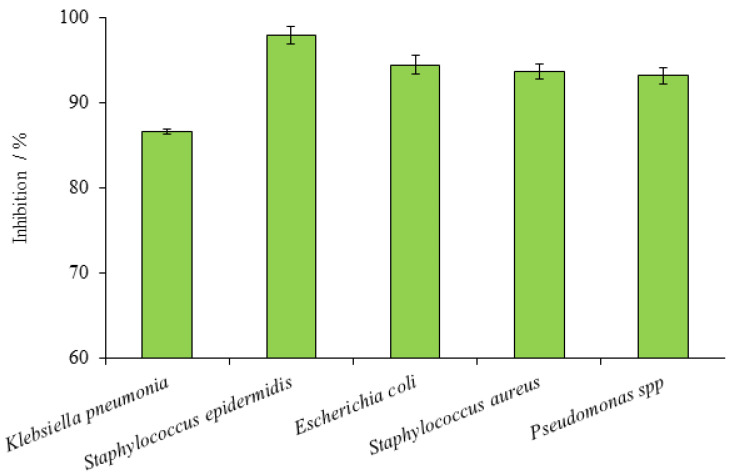
Mean growth inhibition (%) of MEP against five bacterial species. Error bars represent SEM values of results from three replicate experiments.

The MEP was active against the *Klebsiella pneumonia* as well as *Pseudomonas* spp. along with percentage inhibition noted 86.59% and 83.33%, respectively (Figure 4). The mean value of inhibition zone against *K. pneumonia* and *Pseudomonas* spp. on account of MEP was 15.07 mm and 14.73 mm. Susceptibility of these two bacterial strains was observed also against Cefotaxime with 17.40 mm and 17.68 mm zone of inhibition (Table 2). Five selected bacterial species were in combination of both gram-positive and gram-negative strains and outputs reveals significant difference in both groups. Gram-positive strains (*S. aureus* and *S. epidermidis*) were more susceptible to MEP and Cefotaxime while gram-negative strains (*E. coli*, *Pseudomonas spp* and *Klebsiella pneumonia*) were comparatively resistant in accordance with the present investigation.

### 3.3. Antifungal Properties

MEP showed significant antifungal potential against *Alternaria* spp. and *Aspergillus terreus. Alternaria* was comparatively more susceptible than *A. terreus* in the current study against MEP with observed linear growth 22.3 mm in test tube. Whereas measured linear growth was 14.16 mm for fluconazole tested against *Alternaria*. Linear growth was highest in the test tube treated as negative control with 99 mm. The inhibition percentage of *Alternaria* spp. for MEP was observed as 78.45% and for commercial Fluconazole antifungal drug was 86.71% (Table 5). 

The analysis of variance showed that significant differences exist between the two fungal strains tested against MEP and Fluconazole (Table 6).

## 4. Discussions

Results revealed a decrease in value of absorbance with increase in concentration of MEP and AA extract. The decline in value of absorbance of DPPH radical is due to antioxidants, as a result of the reaction between radical progressed and antioxidant molecules, results in radicals scavenging by donation of hydrogen. 2,2-diphenyl-1-picrylhydrazyl (DPPH) is a free radical assay for scavenging an unpaired electron which delocalized over the whole molecule. In this method the change in color was observed from the violet color of the DPPH solution to the yellow colored diphenylpicryl hydrazine product. This is due to addition of the plant sample in a concentration dependent manner which works to determine antioxidant potential. Absorption of DPPH being proportionate to radical concentration being scavenged. Antioxidants respond with free radicals through the mechanism of electron transfer, the antioxidant gives an electron to free radicals and becomes a radical cation. Ionization potential of antioxidant in this mechanism is the significant energetic factor for assessing antioxidant activity. This process has been used widely to predict antioxidant behaviors despite the relatively less time needed for scrutiny. The observed trend is coherent with previous reports in which an inverse relationship was observed between concentration of sample and absorbance value [18]. The radical scavenging potential determined in this assay in terms of inhibition percentage rather gives more comparable results than absorbance [19]. MEP possess a fine ability to scavenge the free radicals, with percentage scavenging ranging from 37.89–63.50% as tested at several concentrations. The 250 µg/mL of AA showed highest observed percentage scavenging i.e., 84.31% while MEP was capable of scavenging free radicals at 63.50% (Figure 1). The results showed the dependence of scavenging potential on the concentration of sample, and a direct relation was observed, so antioxidant potential is directly interrelated with sample concentration. Several other investigations support these result that inhibitory aptitude of all plants strongly interlinked with its concentration [20]. 

The results obtained through PMA facilitate the validation of a direct relationship between absorbance and sample concentration. The phosphomolybdate assay is based on reduction of Mo (VI)-Mo (V) by MEP at acidic pH and successive formation of green phosphate/Mo (V) complex [21]. The sample absorbance signifies the reducing ability and higher value of absorbance is an indication of strong reducing potential, hence it clarifies the dose dependence relationship. It is worth noting that obtained absorbance in the case of MEP was found to be comparable with that of AA with a minor difference explaining the significant level of scavenging capability. The reducing aptitude of the plant in comparison with AA may serve as a significant reflector of antioxidant potential. The obtained outcomes supported in RPA are in confirmation of a direct relationship between sample concentration and absorbance value. RPA is a convenient and fast screening to evaluate reductive capability of MEP. The mechanism of RPA is the capacity of MEP to donate an electron and transformation capability of Fe^3+^ to Fe^2+^, in terms of rising absorbance which increase with concentration [22]. The observed absorbance value reveals the reducing potential of that sample and this may serve as a considerable indicator of its antioxidant activity.

Its antioxidant potential is primarily due to the presence of phytochemical constituent such as bioactive anthocyanin, flavonoids, phenols, and isoflavones [23]. Flavonoid compounds and phenolic components are renowned for their competence and ability to behave as antioxidants. These compounds oxidize free radicals of sample extracts and show standard activity towards less reactive and comparatively more stable radicals. For this reason, flavonoids are considered responsible for stabilizing the ROS through reaction with reactive components of these free radicals. Positive correlation among total polyphenol contents along with antioxidant capability and the considerable amount of TPC and TFC reported in the literature highlight its antioxidant potential. Total phenolic content varied in the range of 489–1273 mg, GAE/100 g and total flavonoid content in the range of 198–3018 mg, QE/100 g [24]. These active phenolic and flavonoid content present in the plant highlights the reason for their reducing ability which is a significant reflector of antioxidant capacity. Hence, the antioxidant capability of the plant may possibly be due to the existence of phytochemicals responsible for the activity.

The susceptibility of the selected *staphylococcus* bacterial strains are rational and coherent with several previously reported outcomes of studies [25]. The *Staphylococcus epidermidis* showed strong susceptibility against *Aquilaria crassna* leaf extract, revealing its considerable antibacterial potential [26]. These findings support the previous outcomes that Lamiaceae family is a very competent contributor to combat skin infection problems caused by highly resistant *S. epidermidis* strains. Synergistic action of plant extracts with essential oil present in members of Lamiaceae family in very significant amounts might be the reason of significant potency against bacterial strains [27]. Hence *Otostegia limbata* may possibly be a remarkable potential source for obstructing particularly problematic skin infections caused by *S. epidermidis*; consequently, further exploration of some of their active compounds should be investigated in the near future.

Regarding the least inhibition showed by various strains of bacteria, the analogous discovery was reported [26] of the susceptibility of various bacterial strains. Various isolates were examined and all isolates were either not or less sensitive to eight antibiotics tested and resistant towards at least one antibiotic commercial drug. Parallel to this report, many other findings also explain the lesser susceptibility and higher resistance of *E. coli* to several tested medicinal plants and many commercial antibiotic drugs [28]. The efficiency of other medicinal plants was also analyzed against these sensitive bacterial strains in various reports which reveal consistent the results with the current investigations [28,29]. Therefore, the above mentioned susceptible two bacterial species were the most, sensitive thus the documented literature is coherent with our current findings. The formerly investigated reports are strongly coherent to the current observation that gram-positive strains are relatively more susceptible, since gram-negative species have an additional outer membrane similar to a covering which guards their cell wall, while others lack this extra cover [30].

*Alternaria* spp. was the most susceptible tested fungal strain in various previously reported studies. Thus, effectiveness of several medicinal plants in previously reported literature against *Alternaria* spp. reveals consistency of these findings.

Fluconazole was strongly active against *Aspergillus terreus* and also sensitive for MEP with observed linear growth for this strain in test tube at 14.16 mm and 32.0 mm, respectively, while linear growth observed against negative control tube was 100 mm. Calculated percentage inhibition of MEP against *A. terreus* was found to be 68% and Fluconazole was 77.33% active against *A. terreus* (Table 4). *Casuarina equisetifolia* was reported as an active medicinal plant with percentage inhibition ranged from 51.78% to 85.80% against *A. terreus,* thus previous reports are in accordance with recent findings (Lagnika et al., 2014). In accordance with current outcomes, *O. limbata* could be a potent source for the control of several fungal infections caused by *A. terreus* and *Alternaria* spp. For this reason, it is recommended that the aptitude of this plant to restrain the fungal strains and bioactive compounds responsible for the antifungal potential be explored further.

## 5. Conclusions

Findings of this study showed that *O. limbata* extract possesses excellent antibacterial, antifungal, and antioxidant potential. The outcomes of the present report showed the considerable antioxidant capability of *O. limbata* as estimated through three assays. Consequently, these results reflect a potent source to avoid and decrease oxidative stresses and age-related diseases including cancer, diabetes, and cardiac diseases etc. Even though further investigations are evidently required to clarify and recognize the bioactive components. The assessment of MEP has shown that some of those were potentially rich sources of antimicrobial agents. However, the maximum potential of the plant was observed against bacterial strains with the highest inhibition percentage. It may be interesting to explore the mechanism of action of the plant against the five bacterial strains tested and also against resistant clinical bacterial strains. These results signify the antibacterial potency of the plant and could serve as a benchmark for the future analysis such as discovery of bioactive compounds responsible for activity against bacterial strains. Thus, this plant could be a natural source of antibiotic drug development which is safer than synthetic medications. 

## Figures and Tables

**Table 1:** Spectrophotometrically recorded absorbance in DPPH assay.

Concentration (µg/mL)	Absorbance (nm)	
	AA	MEP
250	0.153 ^f^ ± 0.044	0.357 ^f^ ± 0.001
125	0.239 ^e^ ± 0.036	0.402 ^e^ ± 0.005
62.5	0.329 ^d^ ± 0.00	0.443 ^d^ ± 0.046
31.25	0.432 ^c^ ± 0.071	0.473 ^c^ ± 0.088
15.625	0.487 ^b^ ± 0.090	0.532 ^b^ ± 0.091
7.81	0.547 ^a^ ± 0.028	0.608 ^a^ ± 0.037

Control absorbance: 0.980; MEP: Methanolic extract of plant; AA–Ascorbic acid; ±–SD of replicates (*n* = 3) (Superscript letters show statistically significant difference determined through Tukey test at *p* < 0.05).

**Table 2:** IC50 value of MEP for antioxidant capacity determined by DPPH, PMA, and RPA.

Sample	IC _50_ Value (µg/mL)		
	DPPH	PMA	RPA
*MEP*	33.01	27.40	56.54
*AA*	15.09	10.79	19.06

**Table 3:** Zone of inhibition against different bacterial strains.

Bacterial Strains	Zone of Inhibition (mm)		
	Cefotaxime	MEP	DMSO
*Klebsiella pneumonia*	17.40 ± 0.40	15.07 ± 0.30	0.00 ± 0.00
*Staphylococcus epidermidis*	17.83 ± 0.15	17.47 ± 0.45	0.00 ± 0.00
*Escherichia coli*	15.10 ± 0.17	12.17 ± 0.15	0.00 ± 0.00
*Staphylococcus aureus*	19.07 ± 0.40	18.80 ± 0.70	0.00 ± 0.00
*Pseudomonas* spp.	17.68 ± 0.73	14.73 ± 0.87	0.00 ± 0.00

±—SD of replicates; Cefotaxime (positive control) 20 mg/mL; MEP: Methanolic extract of plant, 20 mg/mL; DMSO: Dimethyl sulfoxide (negative control).

**Table 4:** Analysis of variance of potential of MEP against the five bacterial strains.

		Analysis of Variance			
	Sum of Squares	df	Mean Square	F	Sig.
**Between Groups**	571.255	4	142.814	9.512	0.002
**Within Groups**	150.133	10	15.013		
**Total**	721.388	14			

(Analyzed using SPSS software, significant difference at *p* < 0.05; Groups represents five different bacterial strains).

**Table 5:** Percentage inhibition against fungal strains.

Fungal Strains	Linear Growth (mm)			Percentage Inhibition	
	MEP	Fluconazole	DMSO	MEP	Fluconazole
*Alternaria* spp.	22.3 ± 1.5	14.16 ± 1.0	99 ± 1.73	78.45	86.71
*Aspergillus terreus*	32.0 ± 1.7	22.67 ± 1.5	100 ± 0.0	68.0	77.33

±—SD of replicates; MEP—Methanolic extract of plant; Fluconazole—Positive control; DMSO—Dimethyl sulfoxide (negative control).

**Table 6:** Analysis of variance of potential of MEP against fungal strains.

		Analysis of Variance				
		Sum of Squares	df	Mean Square	F	Sig.
**MEP**	Between groups	163.804	1	163.804	324.364	0.000
	Within groups	2.020	4	0.505		
	Total	165.824	5			
**Fluconazole**	Between groups	84.600	1	84.600	82.987	0.001
	Within groups	4.078	4	1.019		
	Total	88.678	5			

(Significance analyzed through ANOVA using SPSS software, significant difference at *p* < 0.05; Groups represents two fungal strains).

## Data Availability

The data presented in this study are available upon fair request from the corresponding author.
